# Women's participation on the boards of farmer-owned cooperatives

**DOI:** 10.3389/fvets.2023.1060817

**Published:** 2023-03-30

**Authors:** Henning Otte Hansen, Mette Asmild

**Affiliations:** Department of Food and Resource Economics, University of Copenhagen, Copenhagen, Denmark

**Keywords:** diversity, barriers, external board members, gender, recruitment pool, agriculture, representativeness

## Abstract

Initiatives and specific measures aimed at increasing the presence of women on corporate boards have become widespread. However, not much academic attention has been paid to this subject up till now, when it comes to farmer-owned cooperatives. The article shows that farmer-owned cooperatives do have special problems when it comes to women on boards. The farmer-owned cooperatives in Denmark have been chosen as cases in this article, as they are quite big, exposed to international competition and have substantial market power. Based on annual reports from 25 farmer-owned cooperatives and two of their investor-owned subsidiaries in the years 2005–2022, inputs from present and former board members of farmer-owned cooperatives, CSR-reports etc. a number of conclusions are drawn. Cooperatives have particular challenges with regard to gender diversity on boards due to their specific structure and requirements—compared to investor-owned companies. Different types of barriers that limit women's representation on boards can be identified: (1) Institutional barriers in terms of statutes and cooperative principles. (2) Structural barriers in the form of a narrow or skewed recruitment base. (3) Historical and cultural barriers, where agriculture is typically a male-dominated business. Women's representation on boards of farmer-owned cooperatives is relatively low but increasing. From 2005 to 2021 the weighted average share of female board members has increased from about 1–20%. Gender diversity in farmer-owned cooperatives is consistently less than in listed companies. The increasing representativeness of women is primarily due to the presence of more female external members. Since 2013 the proportion of women has increased, and in 2021 there were more female than male external board members. Female board members are more common in the large farmer-owned cooperatives than in the small. A positive correlation between the size of the companies and the representation of women is identified. This is supported by large cooperatives' greater focus in annual reports and CSR strategies on women's representativeness. Based on the cooperatives' diversity policy, their explicit and specific goals for women's representativeness on boards, interviews with board members etc. a clear awareness of the challenge of gender diversity on the boards is identified.

## 1. Introduction

### 1.1. The relevance of the topic

During the last decades, initiatives and specific measures aimed at increasing the presence of women on corporate boards have become widespread. Norway, in 2005, was the first country to pass a quota law generally requiring at least 40% women on the boards of listed companies (though somewhat dependent on the total number of board members). Subsequently, several other European countries, including Belgium, France, Germany, Iceland, Italy, the Netherlands, Portugal, and Spain have adopted their own board gender quotas. The quotas vary across countries, some with Norway's 40% requirement and others with less (1).

In June 2022, The European Parliament and the EU countries' negotiators finally agreed on a directive to increase the presence of women on corporate boards (2). The aims are to ensure gender parity on boards of publicly listed companies in the EU, and also to ensure that at least 40% of non-executive director posts or 33% of all director posts are occupied by the under-represented sex by 2026.

Small and medium-sized enterprises with fewer than 250 employees are excluded from the scope of the directive. Farmer-owned cooperatives are *per se* also excluded as they are not publicly listed companies. Farmer-owned cooperatives do, however, have special problems when it comes to women on boards:

- According to their statutes, only farmers have the right to become board members, and the overwhelming majority of farmers are men. This means that the recruitment pool for women is relatively small.
- For cultural and historical reasons, there is a tradition for men to have a dominant share of the board positions in farmer-owned cooperatives. This creates a barrier than might be difficult to change.
- The recruitment pool among cooperative owners is continuously shrinking as a consequence of the structural development in agriculture.
- Cooperatives have become larger and more international. The role and responsibilities of the board members—regardless of gender- have increased significantly, which means increasing demands on their specific and diverse skills.

Furthermore, not been much attention has been paid to this subject up till now and not a lot of literature on the topic exists, which may seem surprising given the importance of farmer-owned cooperatives but also the increasing focus on gender diversity.

Therefore we in this article wish to examine the following hypotheses:

- Cooperatives have special challenges with regard to gender diversity on the boards, due to their specific structure and requirements. Structural, cultural, historical, and institutional barriers limit women's representation on boards.
- Women's representation on boards of farmer-owned cooperatives is relatively low but increasing.
- The increase is primarily due to increases in the numbers of female external board members.
- There is a larger share of female board members in the large farmer-owned cooperatives than in the small.
- There is a clear awareness of the challenge of gender diversity on the boards.

The purpose is not to make normative assessments of women's participation in boards of farmer-owned cooperatives. The starting point for the article is that several stakeholders want more women in the top management (employed and elected) of cooperatives, and legislative initiatives also make this topic relevant. From a diversity point of view, the distribution of board members is skewed which, from a purely business perspective, is also a possible sign of inefficiency. The hypothesis above are analyzed based on observational cross-sectional as well as longitudinal data and a positivistic research philosophy.

Women's participation in cooperatives can take place through several forums: through the board of directors, board of representatives or general meeting, through committees and working groups set up by the cooperative or through ongoing discussion, exchange of ideas and constructive dialogue with the cooperative as shareholder. The concept of active ownership in cooperatives is widespread, and the intention is to activate all members in the discussion about the operation and development of the cooperative. Strategies for the cooperatives must be decided by the board of representatives, but the board—supported by the management—is typically the executing party and the central player when it comes to strategies and future development of farmer owned cooperatives. In this research we focus on women's participation on the boards of farmer-owned cooperatives, while women's participation in other forums is left for further research.

For several reasons, the conditions and the farmer-owned cooperatives in Denmark have been chosen as cases in this article: Farmer-owned cooperatives are rather common and important in the Danish agricultural and food industry, which is furthermore highly developed and exposed to international competition. Some of the farmer-owned cooperatives in Denmark are among the largest in the world within its business segment, demanding a high degree of competencies and leadership from the owners. Furthermore, information about board members of Danish companies is readily available.

It is, however, assumed that the identified problems, hypotheses and possible solutions are relatively generic and relevant for farmer cooperatives in most countries, perhaps especially the most economically developed ones.

### 1.2. Farmer-owned cooperatives: Uniqueness, relevance, characteristic etc.

This article focuses on farmer-owned cooperatives alone. Farmer-owned cooperatives have substantial differences compared to, for example, investor-owned companies. Especially in relation to women's participation on the boards, special conditions apply (as described below), which can constitute significant barriers, making it pertinent to focus on this selected business type.

There is no universally accepted definition of a cooperative. In general, a cooperative is a business owned and democratically controlled by the people who use its services and whose benefits are derived and distributed equitably on the basis of use. The user-owners are called members.

The International Cooperative Alliance (ICA), defines a cooperative as “an autonomous association of persons united voluntarily to meet their common economic, social, and cultural needs and aspirations through a jointly-owned and democratically-controlled enterprise” (3).

Another widely accepted definition is that “A cooperative is a user-owned, user-controlled business that distributes benefits on the basis of use” (4). This definition captures what are generally considered the three primary cooperative principles: User ownership, user control, and proportional distribution of benefits.

Farmer-owned cooperatives often base their regulations on the seven international cooperative principles established by the ICA:

- Voluntary and Open Membership.
- Democratic Member Control.
- Member Economic Participation.
- Autonomy and Independence.
- Education, Training and Information.
- Co-operation among Co-operatives.
- Concern for Community.

Several of these principles are important when it comes to women's participation on the boards of farmer-owned cooperatives.

Cooperatives are democratic organizations controlled by their members, who actively participate in setting their policies and making decisions. This usually means that the members themselves personally join the boards. As a consequence, the farmers themselves enter the board of farmer- cooperatives, which significantly limits the diversity of the board's recruitment pool.

**Figure 1:**
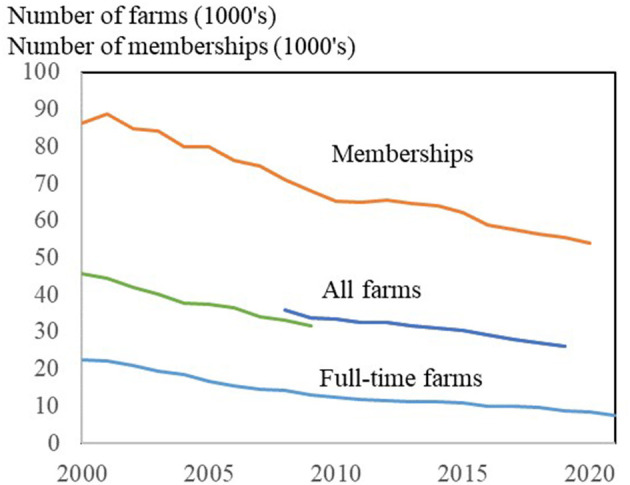
Development of farms and farmer-cooperative memberships in Denmark. All farms: Discontinued time series 2008–09. Source: Own calculations based on Statistics Denmark (5) and annual reports from companies and organizations.

The limited diversity is further reinforced by the fact that the number of farmers, and thus the number of directly eligible members of the cooperatives' boards, decrease over time. As shown in Figure 1, the number of farms in Denmark has decreased by almost 50% since 2000, and especially the number of full-time farms has decreased.

During the period of 2000–2020, the number of memberships of farmer-owned cooperatives has also decreased significantly. It is worth noting that the number of memberships is greater than the number of farms. The explanation is that a farmer is typically a member of several cooperatives.

Cooperatives are by definition autonomous, self-help organizations controlled by their members. Members are thus numerous and unconcentrated, as all members have equal voting rights (one member and one vote).

However, the cooperative may also choose board members outside the ownership group. This is typically the case if individuals with special leadership competences etc. are required. In these cases, which are becoming more and more common, access to professional competences, experience and, not least, complementarity are important characteristics.

Unlike cooperatives, the owners in investor-owned companies have influence in the sense of one dollar, one vote. Voting rights depend on the amount of money invested *via* the number of shares. Investors can appoint other people to the boards. As long as they comply with specific competence requirements and are fit-and-proper, there are few restrictions when it comes to appointing people to participate in board work, i.e., regarding nationality, gender, etc. Therefore, the pool for recruitment is far broader than in cooperatives. Conversely, the board members of a cooperative often have a very keen interest in the company, and have strong personal (economic) incentives for ensuring a profitable and long term development of the company.

Overall, the conclusion for now is that cooperatives and investor-owned companies of course have some similarities but also clear differences. Both types of companies develop over time and thus it is a recurrent feature that modern cooperatives, in many cases, have developed so that they share many features with investor-owned companies. Generally, there has been a tendency for cooperatives to increasingly focus on business, while non-economic, non-commercial and ideological aspects have become less important. In addition, several hybrid models have developed which are crosses between cooperatives and investor-owned companies, or which contain both cooperatives and investor-owned companies in the same company.

Cooperatives and investor-owned companies are therefore far from unambiguous company structures and there can be large differences between different types of cooperatives, and also big differences from country to country. Despite this, it is possible to identify a number of general and important similarities and differences between cooperatives and investor-owned companies (6).

The prevalence of farmer-owned cooperatives varies considerably from sector to sector and from country to country, which can partly be explained by the different market conditions, which to a greater or lesser extent stimulate the need for—and the benefits of—the cooperative organization. Specifically for cooperatives owned by farmers, it is evident that cooperatives are most widespread in North America, Northern and Central Europe, as well as in Japan and Korea.

In general, cooperatives, regardless of sector and industry, are most important in the most economically developed countries. Cooperatives in these countries have a relatively large market share and many farmers are typically members of one or more cooperatives. An important explanation for this different importance between countries is that the establishment and management of cooperatives requires a certain level of infrastructure, training and organization, which is not always present in the less developed countries.

When it comes to differences between industries, the cooperative organization is particularly common in the supply and processing activities that are closely linked to agricultural production in the value chain, i.e., downstream, whereas farmer-owned cooperatives are rarely found in activities close to consumers. This producer- rather than consumer-orientation may also be a factor in women's representation on boards of farmer-owned cooperatives.

### 1.3. Farmer-owned cooperatives in Denmark: Management challenges

The purpose of this section is to illustrate and document that the farmer-owned cooperatives in Denmark are quite big and exposed to international competition, which in turn means that management, tasks and responsibilities are quite demanding and business oriented.

That the Danish farmer-owned cooperatives have substantial market power and faces international competition is evident from Table 1 below, which shows the market shares for farmer-owned cooperatives in Denmark in the major agri-industrial industries.

**Table 1:** Farmer-owned cooperatives' shares of their respective markets in Denmark in 2021 (or latest year with available data).

**Product**	**Percent**
Whole milk deliveries	92
Butter	99
Cheese	92
Pork	72
Beef	63
Grass seed	76
Egg	40
Sugar	0
Poultry	0
Agr. machinery	0
Fruit and vegetables	53
Feed and fertilizer	80
Potato starch	100

Pork and beef: Share of slaughtering.

Eggs: The major shareholding egg company owned by farmer cooperatives is not included.

Fruit and vegetables: Fresh products for direct consumption.

Feed and fertilizer: Companies producing feed and selling fertilizer.

Source: Own calculations based on Hansen (7), Danish Agriculture and Food Council (8–10), and annual reports from companies and organizations.

Table 1 shows large market shares for farmer-owned cooperatives within the dairy and meat industry. The grass seed and the potato starch industries have gained a very strong competitive position, where the cooperative structure has been an important competitive strength (11).^1^

Contrary to this, the cooperative model has failed within the sugar, poultry and agricultural machinery industries. Cooperatives did exist in these industries, but for different reasons they could not compete, they failed or became unnecessary, and the cooperatives were subsequently acquired by other investor-owned or foreign companies.

The very different market shares for cooperatives from industry to industry can also be explained by the fact that the cooperative ownership has both advantages and disadvantages, which can have varying importance depending on structure, value chain, internationalization, capital intensity, etc. For example, cooperatives are common in the dairy sector around the world. The explanation is, that farmers who have daily production of fresh and perishable agricultural products, have a great incentive to secure right of delivery and a stable buyer (12).

**Figure 2:**
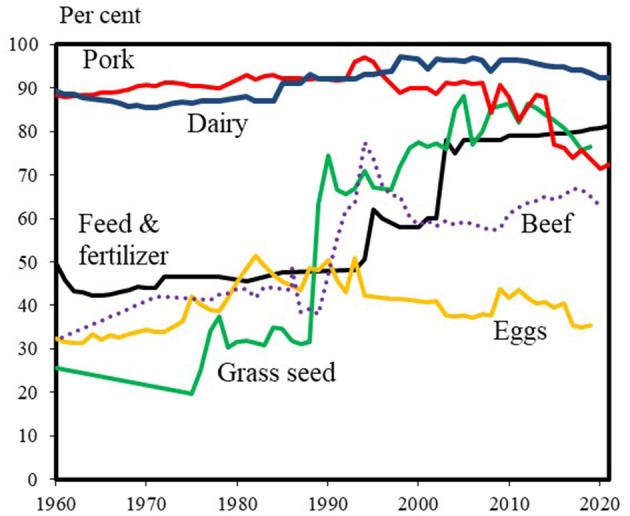
Domestic market shares for agricultural cooperatives in selected industries. Source: Own calculations based on Danish Agriculture and Food Council (8–10) and annual reports from companies and organizations.

Cooperatives have existed in Denmark since the 1880's. Cooperatives initially arose in the dairy and meat industry and subsequently spread to other agricultural branches (6). As illustrated in Figure 2, the cooperative market shares within Denmark have generally been stable in the recent decades, and growth has been achieved through foreign activities. This shows that the cooperative model has been viable and competitive in a period characterized by liberalization, globalization, and consolidation.

The increasing market shares for the feed and fertilizer industry is mainly due to acquisitions of investor-owned competing companies. To a certain extent, the acquisitions were a result of poor management of the acquired investor-owned companies, which was an important factor in why they were sold. This supports the fact that management is an important competitive parameter in these industries as well.

For many decades, most agricultural industries in Denmark have been strongly export-oriented—typically with export shares of 70–90%. More recently, growth has increasingly taken place through investments, production and establishment in foreign countries. This form of internationalization requires additional strategic business management. Internationalization through foreign direct investments, global strategic alliances and global mergers, which have been a massive trend in recent decades, are often difficult for cooperatives to cope with due to their structure and form of ownership: When the overall goal is to ensure the owners—the farmers—attractive sales prices and/or lower input prices, investments in foreign companies, sourcing of agricultural products from foreign farmers etc. can be perceived as irrelevant or aimless for the owners.

All in all, the Danish farmer-owned cooperatives are large and to a considerable extent engaged in international activities, as is illustrated in Table 2.

**Table 2:** Top-6 Danish farmer-owned cooperatives: Turnover and foreign activities.

		**Turnover**	**Share of foreign activities**	
		**Company**	**Business**	**Euro bn**	**Percent**	
Arla Foods amba	Dairy	11.2	64	Share of milk intake outside home country
DLG amba	Farm supply	7.9	71	Turnover from foreign activities
Danish Crown amba	Meat	7.8	63	Employment abroad
Danish Agro amba	Farm supply	5.9	60	Turnover from foreign activities
DLF seeds	Grass seed	1.0	60	Assets in foreign countries
KMC amba	Potato starch	0.3	94	Export share

Amba means “cooperative with limited responsibility”.

Arla Foods is a transnational cooperative with members in seven different countries but has its headquarter in Denmark and is formally registered in Denmark.

Source: Own calculations based on annual reports of the companies.

Table 2 shows selected key figures for the six largest farmer-owned cooperatives in Denmark. The respective estimates for the shares of foreign activities are also noted. Most of these companies are among the largest in Europe, or in the world, within their specific business area. This emphasizes that the needs for strategic management, and thus the requirements for the board members, are high.

### 1.4. Literature review

Gender diversity and women's representation in farmer-owned cooperatives is not a heavily analyzed topic. Topics such as gender diversity in business, diversity on corporate boards, management challenges in farmer-owned cooperatives etc. are well-analyzed, whereas the specific combination of the topics is much less studied.

Phil Kenkel studies board diversity in agricultural cooperatives, and underlines, that data on the board composition in agricultural cooperatives is limited (13). However, data from United States shows, that females make up just over 3% of board members in agricultural cooperatives—the lowest representation of any cooperative sector. It is concluded, that agricultural cooperatives clearly trail other cooperative sectors, as well as investor-owned firms, when it comes to board gender diversity.

Kenkel also touches upon the fact, that an increasing number of cooperatives are implementing an associate board structure as a way to increase diversity (13).

Aazami et al. analyze women's level of participation and the factors influencing their involvement in different stages of cooperative activities in Shiraz, Iran (14). The study concerns a women's cooperative and does not relate specifically to women's representation in farmer-owned cooperatives or to gender diversity. The analysis concludes, among other things, that women's participation was mostly at the level of “surrender” or “acceptance.”

Women's active participation in cooperatives—as ordinary members or as members of the board, respectively—has some common barriers and limitations. A formal election to a board, including support from members, possible election campaign, etc., involves greater cultural and human barriers. Further, measures of gender representation typically relate to the board and not to general participation.

Food and Agriculture Organization of the United Nations (FAO) investigates agricultural cooperatives and gender equality, and notes that rural women have less access to the resources and opportunities in agriculture than men (15). FAO also recommends governments and international organizations to implement policies that foresee quotas or targets for women's participation in cooperative boards.

International Labor Organization (ILO) describes a survey about the relationships between the cooperative movement in general, women's empowerment and gender equality (16). Fifty percent of the survey respondents were from Europe, 15% from Asia, and 15% from North America. The study concludes, that “women are among the most involved in and served by co-operative organizations, but among the least likely to hold high-ranking and decision-making roles” (16). When it comes to agriculture, the survey results suggest that women's participation in leadership in the agricultural sector in all regions of the world is significantly below the average for other sectors.

## 2. Materials and methods

The following five types of material and data have been considered for this article:

- Annual reports from 25 farmer-owned cooperatives (see Appendix) and two of their investor-owned subsidiaries in the years 2005–2022 (around 450 annual reports).

° Purpose: Access to information about board members (number of members, member groups, and gender).

- Homepages from farmer-owned cooperatives.

° Purpose: Access to information about board members (member group and gender) in present boards not yet publishing annual reports.

- CSR reports from farmer-owned cooperatives when available.

°Purpose: Access to information about targets and goals for gender diversity.

- Relevant literature about or related to the topic of this article.

° Purpose: To uncover existing literature in order to compare issues and solutions with other studies.

- Comments, statements, assessments from present and former board members of farmer-owned cooperatives.

° Purpose: To have access to deeper information about barriers, drivers, motivations and attitudes regarding female participation in farmer-owned cooperatives.

Whilst the data primarily consists of annual reports from farmer-owned cooperatives, it is important to note that in two special cases data from investor-owned subsidiaries is also included. Since this impacts the subsequent results, this choice is explained and justified as follows.

In the subsequent analysis of gender diversity in farmer-owned cooperatives, the diversity is estimated both in a group consisting exclusively of cooperatives, and in a group where cooperatives' farmer-owned subsidiaries replace the parent cooperative. This is because a few large cooperatives have established investor-owned subsidiaries during the study period, which are wholly or almost wholly owned by the cooperative. This company construction is typically established for two reasons: First, by setting up an investor-owned subsidiary, the company is prepared for a situation where it is desirable to attract external investors as shareholders. Secondly, the purpose may be to make room for external board members and thus new management competence in the company. Often, the statutes of the cooperative will be a barrier to both external capital and external board members, which is why an investor-owned subsidiary can be an appropriate solution.

The model with the establishment of subsidiaries is only used where the most important strategic management takes place in the investor-owned subsidiary and where the cooperative is a majority shareholder. In this article, this is the case for two companies: DLF amba and Danish Crown amba.

DLF amba is a seed cooperative dealing in forage and amenity seeds, and other crops. DLF amba has for a long time almost fully owned the subsidiary DLF A/S, which is a limited company. Until 2021, DLF amba only had cooperative members (farmers) on the board, while DLF A/S, on the other hand, had a long tradition of having external members. From 2017, there was also a female external member of the board of DLF A/S. From 2021, the structure has changed, so that the board of DLF amba has been expanded and now also has external members, while the board of DLF A/S has been reduced and now only has cooperative members on the board.

Considering the dominant ownership of the cooperative in the subsidiary, we here consider the members of the boards of the limited subsidiary until 2020 and of the cooperative from 2021. In parallel with this, the members of the board of directors in the cooperative throughout the period are also included in the estimations.

Danish Crown amba is a food manufacturing cooperative in Denmark, dealing primarily in meat processing of pork and beef. It is Europe's largest pork producer. In 2006, the cooperative introduced two external board members, but in 2013 they were transferred to the 100% owned subsidiary, Danish Crown A/S, which is an investor-owned company. Since then, the number has increased to 4, and in 2022 50% of the board members are women. In the cooperative parent company, Danish Crown amba, only cooperative members are elected to the board.

We here count the members of the boards partly in the cooperative, partly in the cooperative's subsidiary with external board members depending on where the most important strategic decisions are made.

The cooperative dairy company, Arla Foods amba, is a special case: In 2019 the company appointed two external board members, in the beginning without the right to vote. From 2022, they have become full members with full voting rights. So whilst they do not have investor-owned subsidiaries, Arla Foods amba has still allowed for external members on the board. Throughout the period, only female external members have been appointed. We here include the external members in the estimations for the entire period, regardless of voting rights or not.

Regarding the choice of method, the empirical basis is not sufficient to carry out econometric or statistical analysis. The analyses will therefore be based on the collection and processing of primary data, descriptive statistics, as well as qualitative studies, which is considered to be the optimal method for investigating the topics of this article.

## 3. Results

Based on the material and the methods described in the previous section, a number of interesting results have been obtained, which can be used to confirm or reject the established hypotheses.

**Figure 3:**
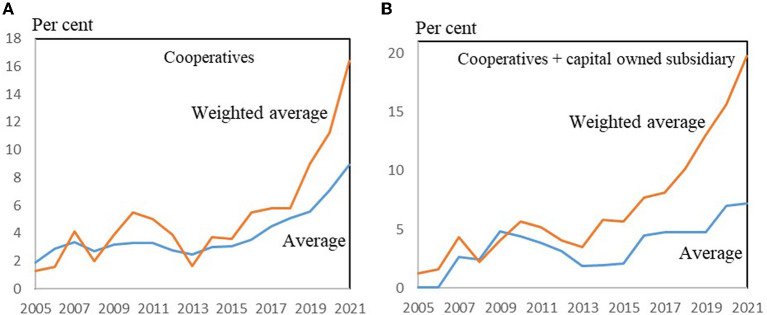
Female board members (share of all board members) of Danish farmer-owned cooperatives, 2005–2021. **(A)** Average of 25 farmer-owned cooperatives. **(B)** Cooperatives + cooperatives with a shareholding subsidiary with external members. Source: Own data collection, calculations and presentation based on annual reports and homepages of 25 farmer-owned cooperatives in Denmark.

In Figure 3, the overall trend for female representation on the boards since 2005 are shown for both the cooperatives (A) and for the cooperatives + investor-owned subsidiaries (B).

In Figure 3, the “Average” means a simple average of the number of female board members as a percentage of all board members in the 25 farmer-owned cooperatives. For the “Weighted average” the share is weighted by the size of the companies as indicated by the turnover. The 25 cooperatives are of very different sizes, ranging from 20 million Euros to 10 billion Euros in annual turnover in normal years.

In Figure 3 the trend for female representation on the boards since 2005 is relatively clear, regardless of whether only cooperatives (A), or cooperatives + investor-owned subsidiaries (B) are considered: women's shares on the boards of cooperative agricultural companies is increasing, and especially in the last 5–10 years a significant increase can be seen.

The boards under B are only considered in cases where the most important strategic management takes place in the investor-owned subsidiary and where the cooperative is a significant majority shareholder (c.f. Section 3 above). Women's average representation on the boards is larger when these subsidiaries are also considered, as can be seen when comparing the left and the right panel of Figure 3.

Figure 3 also shows that the weighted shares are considerably larger than the unweighted shares, and that the differences are increasing. It shows that women's representation is larger in large companies. The increase in women's representation has mainly occurred in large companies.

**Figure 4:**
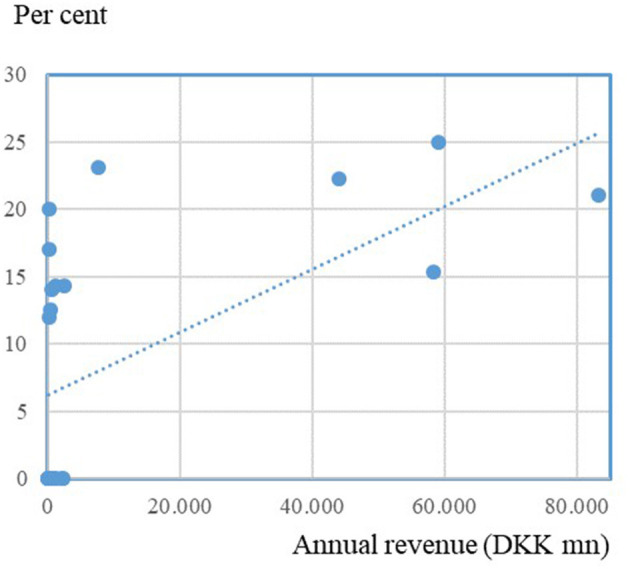
Size of farmer-owned cooperatives and share of women on the boards. Source: Own data collection, calculations and presentation based on annual reports and homepages of 25 farmer-owned cooperatives in Denmark.

To investigate this pattern further, Figure 4 illustrates the relationship between the size of the companies and the share of women on the boards of the individual companies.

Figure 4 shows a positive correlation between the size of the companies and the representation of women, where we see that the largest cooperatives have the largest shares of women on their boards (15–25%). The small companies are more varied, with between 0 and 23% women on their boards. The correlation is also supported by the greater focus on diversity in the large cooperatives' annual reports. Note that we do not here argue causality, nor hypothesize on the direction of any possible causality between size and female representation, as any casual relationships between board composition and companies' performance are usually difficult to verify.

Specifically regarding female board members and based on Norway's relatively long-term experience, Eckbo et al. conclude, that mandatory board gender-balancing did not reduce firm value or performance significantly (1). Another study concludes, that the average effect of gender diversity on firm performance is negative (17).

Board membership for the farmer-owned cooperatives takes place in three membership categories: Cooperative members (farmers), employees, and external members.

Cooperative members are elected in accordance with the statutes among the active members of the cooperative. Typically farmers, or managers on larger farms, can vote and can be elected. Each farmer or farm has one vote.

The employees also have the right to be represented on the boards according to the present legislation, however this depends on the company's size and form of ownership. In companies with at least 35 employees, the employees have the right to elect a number of board members corresponding to half of the other members (18).

Finally, several cooperatives have now introduced a third group of members, external members. In these cases, the statutes allow the general meeting and/or the board members to give external persons a seat on the board.

The starting point is that all board members have the same influence and responsibility, so it is reasonable to consider all members of the board as one, regardless of member group. This is the assumption when assessing women's influence and representativeness. It should be noted, however, that cooperatives and their management have no influence on whom the employees choose for the boards.

**Figure 5:**
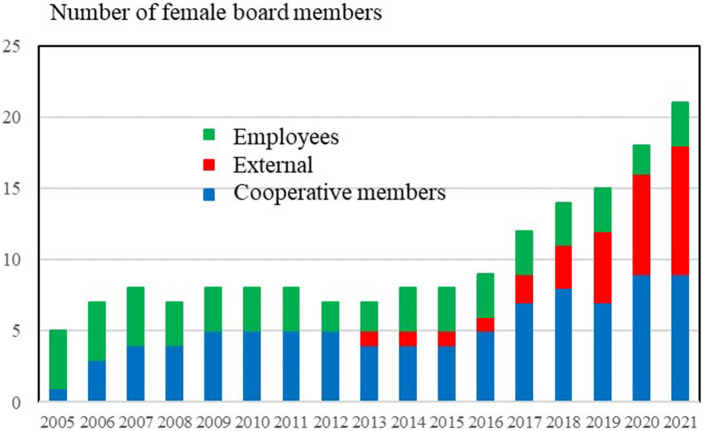
Groups of female board members of Danish farmer-owned cooperatives, 2005–2021. Source: Own data collection, calculations and presentation based on annual reports and homepages of 25 farmers owned cooperatives in Denmark.

Figure 5 shows the total number of women in farmer-owned cooperatives 2005–2021 for each member group.

Figure 5 shows that the increasing representativeness of women is primarily due to the presence of more female external members. Women first appeared as external board members for the agricultural cooperatives in 2013, and since then the number has increased to around 43% of all women on the boards in 2021. The number of female cooperative members on the boards is increasing, but at a slower pace and from a relatively low level. Approximately 9% of cooperative board members in 2021 were women.

Among the employees, women's representation has been rather stable, and they make up a relatively small proportion of the total number of women on the boards (14% in 2021). In contrast, female external members are now as large a group as female cooperative members.

Women's increasing representation *via* the role of external members can be seen as a result of the cooperatives' initiatives to increase women's representation within existing statutes: With a small recruitment pool, it is difficult to increase the proportion of female cooperative members on the boards. Given these limited options, the appointment of female external members is a relatively easy way to increase women's representation on boards.

**Figure 6:**
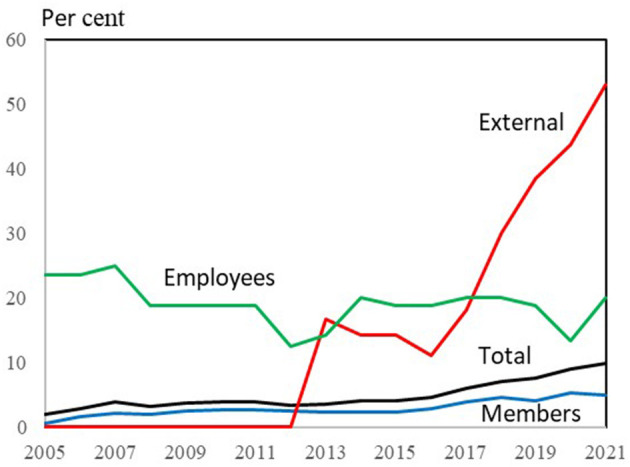
Board member groups of Danish farmer-owned cooperatives, 2005–2021: Share of female board members. Source: Own data collection, calculations and presentation based on annual reports and homepages of 25 farmers owned cooperatives in Denmark.

The development in women's shares of board members in the various member groups is also illustrated in Figure 6.

Figure 6 shows both the level and the development of women's representation in the various member groups. The employees contribute to a higher total share throughout the period, while external members contribute a lot in the last part of the period. In 2021, women made up only 5% of the member-elected board members.

**Figure 7:**
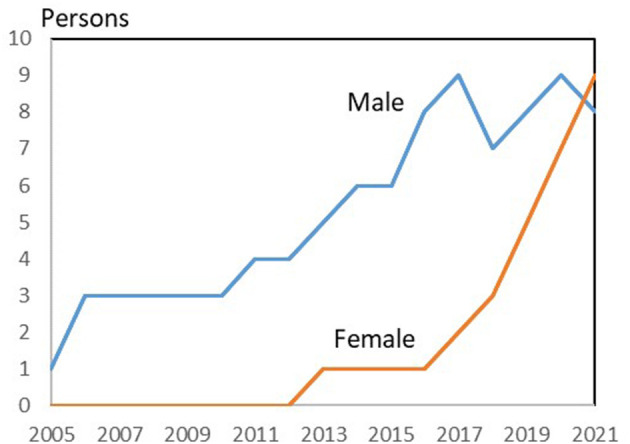
External members of Danish farmer-owned cooperatives: Male and females. Source: Own data collection and presentation based on annual reports and homepages of 25 farmers owned cooperatives in Denmark.

The increasing representativeness of women *via* external members is evident from Figure 7, which shows the male and female share of external board members of Danish farmer-owned cooperatives.

The figure shows that male external board members have existed throughout the period, while women have only been appointed as external members since 2013. Since then, the proportion of women has increased, and in 2021 there, for the first time, were more female than male external board members.

**Figure 8:**
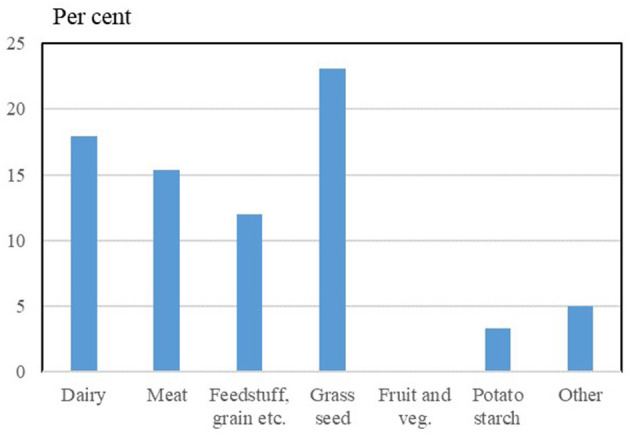
Share of women on boards of farmer-owned cooperatives in different sectors (2021). “Other” includes primarily fur-feed cooperatives and an egg wholesale cooperative. Source: Own data collection and presentation based on annual reports and homepages of 25 farmers owned cooperatives in Denmark.

Women's representation on cooperative boards probably varies from sector to sector. Some sectors may be more or less male-dominated based on e.g., historical reasons. The differences in women's shares of board positions in different sectors are shown in Figure 8.

However, the differences between the sectors must be interpreted with caution, as the structure in the individual sectors is very different. The sector “meat industry” thus consists of only one large company, while the group other “industries” consists of many but relatively small companies.

Companies, regardless of ownership but of a certain size, are obliged to set targets and present policies for the underrepresented gender, and report on this. Several cooperatives thus have more or less explicit and concrete targets for gender diversity on the boards. It ranges from rather vague intentions to specific goals with both numbers and times specified. Concrete action plans can also be included in companies' diversity policy in their CSR reports.

**Figure 9:**
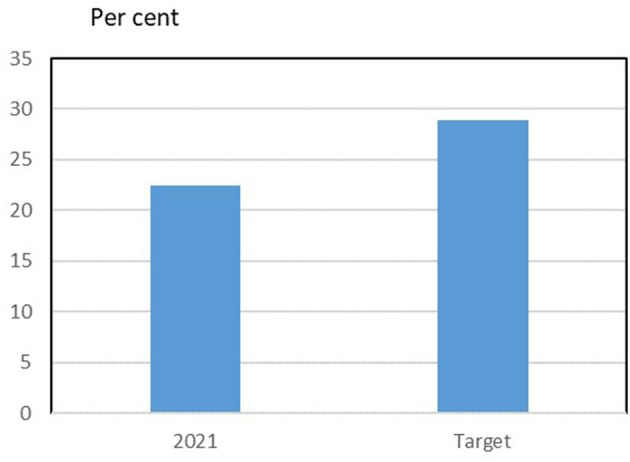
Female board members (present and target) of Danish farmer-owned cooperatives. Includes five farmer-owned cooperatives with explicit and quantitative target for share of female board members. Targets refer to cooperative members, and external and employee members are assumed to be unchanged. Targets are expected to be met in different years in the period 2004–2030. Source: Own data collection and presentation based on CSR- reports and homepages of farmers owned cooperatives in Denmark.

Explicit goals or targets for women's representation on the boards of the cooperatives for the whole farmer-owned cooperative industry are, however, difficult to quantify when the degree of specificity is so different, has different time horizons, and sometimes is completely non-existent. Based on data from the cooperatives with rather explicit goals for gender diversity, the potential for women's further representativeness on boards can be calculated, and are illustrated in Figure 9.

Companies' goals, ambitions and intentions regarding gender diversity cannot be used uncritically as an estimate for future development. However, the figure shows that the targets are above the current level. Still, the goals do not reflect full gender equality, so even if the goals are met, women will be underrepresented. However, the goals can be said to be realistic and not an expression of a long-term ambition.

## 4. Discussion

The analysis and the results found in the previous chapters have led to new issues, questions, discussions and perspectives arising. Several barriers have been identified and revealed, and the recruitment pool for cooperatives seems to be a consistent theme, and the problems with the recruitment of more women on the boards may be almost chronic and intractable for the cooperatives in several ways:

- The number of cooperative members is decreases continuously, and thus the recruitment basis among members becomes smaller. The recruitment pool among potential female board members will therefore also be smaller, which—ceteris paribus—makes the companies more vulnerable.
- Women already make up a very small proportion of the members of the cooperatives.
- Historical, institutional, and cultural barriers seem to limit women's share of the recruitment pool in agriculture. The persistent low number of women in agriculture and limited opportunities for external members according to statutes make it difficult to increase gender diversity significantly.
- The statutes and the business ideas of farmer-owned cooperatives idea are based on the premise that farmers participate actively and elect board members among themselves. The options for female external board members exist, and are used, but have limited potential. External board members should not have a dominant role in the boards, if the principles in cooperatives are to be followed. The farmers and the members will probably also be very reluctant to hand over the majority influence—or just a significant influence—on the boards to external members, cf. for example an interview with a former president of the Danish Agriculture Council, Peter Gæmelke (19).

The rather modest recruitment pool is a difficult barrier. The question is whether the barriers are structural and long-term, and whether they are expected to continue.

In Denmark, on which this study is based, female farmers own only 5% of the agricultural land, while men own 81%, and the rest is owned by companies (20). The share has been rather constant between 2010 and 2019 (21). Approximately 20% of the employees in agriculture, forestry and fishing together are women (21).

Men also make up the dominant proportion of members of cooperatives, of their representatives, and of the delegate assembly (Table 3).

**Table 3:** Women's shares (percent) of members in farmer-owned cooperatives (2022 or recent years).

**Cooperative**	**Percent**	**Recruitment pool**
Arla Foods amba	14	Farmer-elected members of the board of representatives
Danish Crown amba	3	Farmer-elected members of the board of representatives
DLG amba	15	Farmer-elected members of the board of representatives
Danish Agro amba	10	Delegate assembly
DLF amba	2	Delegate assembly
AKK amba	<2	Members
AKM amba	4.5	Members
AKS amba	1.6	Members

Source: Own presentation based on CSR-reports from the individual cooperatives.

Definitely, the recruitment pool of potential female board members among farmers is modest, and it will stay low for a long period due to structural, traditional and historical reasons, since the number of farmers and farms is continuously decreasing, and women consistently constitute a very small proportion of them. These structural conditions seem to be quite persistent.

The chairman of a farmer-owned cooperative with a very low number of female members, Karup Kartoffelmelsfabrik (AKK amba) himself considers that a certain gender diversity in a cooperative's board and management is to the advantage of the company, ceteris paribus. This is also something the company both strategically and specifically works for. However, the recruitment pool is a significant barrier. As an example, typically a maximum of five out of ~125 participants at the general meeting in Karup Kartoffelmelsfabrik are women, and in that forum members to the board of directors are elected. This corresponds to 4%, compared to the current proportion of women on the board of 14%.^2^ As shown in the Table 3, <2% of the farmers or members are women.

Other board members in farmer-owned cooperatives also indicate the small recruitment base as one of the most important barriers to increased gender diversity on their boards. According to a farmer with long-term experience as a board member and chairman in a number of both farmer owned cooperatives and investor owned companies, the relatively small share of women in agriculture is a significant institutional explanation. However, other factors are also important: It turns out that once women constitute a certain proportion of the elected members, it becomes easier to attract additional women to the boards. Legitimacy increases the more women are elected. Several other cultural barriers also exist, but a focused and persistent effort can reduce them.^3^

Culture and traditions are also highlighted as specific barriers for women on boards. For example, the chairman of Arla Foods emphasizes that it is a question of culture when less women are represented at the top of the cooperatives in Denmark than in e.g., neighboring Sweden (22). He points out that influencing and changing the culture in agriculture in order to improve gender diversity is probably a very difficult challenge.

The specific initiatives to attract more women to the boards vary greatly in form and scope from cooperative to cooperative. An example is DLG amba, which in recent years has increased the number of both external and member-elected female board members. Since 2019, DLG has thus increased the proportion of women on the board (cooperative members and external members) from 0 to 23%. As a specific measure to achieve the goals of greater female representation, DLG had advertisements in the agricultural media and held an inspiration meeting (23).

Arla Food, which has also increased the representation of women on the board in recent years, has no concrete initiatives to attract more member-elected women to the board. According to the chairman, the ambition to have more women on the board of representatives is achieved by formulating and explaining the issue in the relevant fora (22).

Initiatives such as special quotas have also been discussed in the cooperatives. The chairman of DLF amba emphasizes that he would like to see more women on the board, but no plans for quotas or other active measures to get more women on the board have been suggested or implemented. The reason is that the members are elected in constituencies where they elect the delegates. It is a democratic system that works as intended, and qualifications are given the highest weight (24). It shows a potential conflict between the perception of democracy and diversity.

As has been shown previously, in recent years gender diversity on average in Danish farmer-owned cooperatives has improved substantially. The questions are whether a reasonable level of diversity has now been ensured, and whether an independent gender diversity problem in farmer-owned cooperatives exists. However, the average figures cover a considerable spread: The figures for the individual companies show, that half of the cooperatives in this survey have no women on the boards at all.

Several large cooperatives have presented diversity goals which show that there is still a need for more women on the boards in order to achieve the goals, some of which are even quite short-term.

Even with the presented diversity goals, there is a long way to full gender diversity. In reality, coming close to something resembling full equality, which may not be a target, is probably impossible, as the share of external board members hardly can be increased substantially any more, as statutes prevent new access. Furthermore due to the cultural and historical barriers, the number of member-elected women in the boards is likely to be relatively permanent.

**Figure 10:**
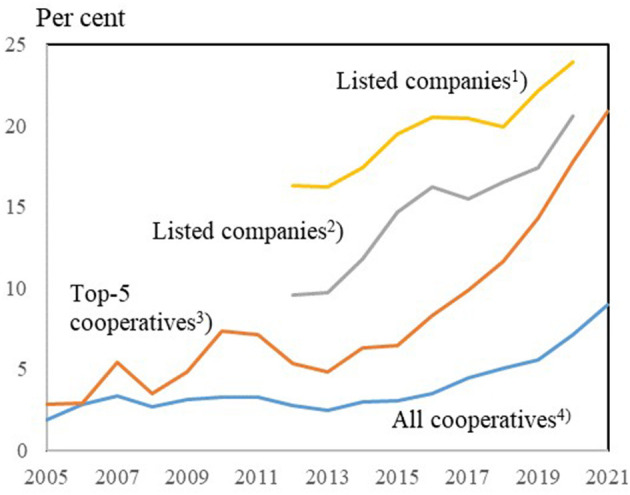
Women's shares (percent) of all board members in different types of companies in Denmark. ^1^Elected by the general meeting and by employees in listed companies. ^2^Elected by the general meeting. ^3^Elected (total) in farmer-owned Danish farmer-cooperatives: Five biggest cooperatives. ^4^Elected (total) in farmer-owned Danish farmer-cooperatives: All cooperatives. Sources: Own calculation and presentation based on (25) and annual reports from cooperatives.

Compared to other industry sectors, it appears that farmer-owned cooperatives have—or have had—a gender diversity problem of their own. As Figure 10 shows, gender diversity in farmer-owned cooperatives is consistently less than in listed companies.

Figure 10 shows a general and significant increase in share for all four groups of companies. The five largest farmer-owned cooperatives, which most of all can be compared to listed companies, have since 2015 increased the representation of women on the boards so much that they are now almost at the level of listed companies. For all farmer-owned cooperatives on average—and thus in particular for the smaller farmer-owned cooperatives—the diversity gap is still significant.

The increased proportion of women on the boards of farmer-owned cooperatives is primarily, and almost exclusively, due to more external female board members, i.e., non-members and thus non-farmers. Typically, the purpose has been to supplement the boards with competencies that did not exist to a sufficient extent among the member-elected board members. An interesting characteristic is the fact that the proportion of women is far greater among the external members than among the member-elected members. However, in a democratically led company, it is also important that the owners are well-represented and that they have the necessary influence to ensure that the company develops according to the purpose. A balance must be ensured: On the one hand, the board must be anchored among the owners to ensure both legitimacy and support. On the other hand, external female board members can ensure both better gender diversity and access to necessary management competencies on the board.

## 5. Conclusion

Although the discussion identifies or exposes new barriers and issues, the hypotheses set out at the beginning of this article can all be confirmed based on the analyses, results, and discussions.

Cooperatives have particular challenges with regard to gender diversity on boards due to their specific structure and requirements, compared to investor-owned companies. Different types of barriers that limit women's representation on boards can be identified:

- Institutional barriers in terms of statutes and cooperative principles.
- Structural barriers in the form of a narrow or skewed recruitment base.
- Historical and cultural barriers, where agriculture is typically a male-dominated business.

Women's representation on boards of farmer-owned cooperatives is relatively low but increasing. From 2005 to 2021 the weighted average share of female board members has increased from about 1 to 20%. Gender diversity in farmer-owned cooperatives is consistently less than in listed companies.

The increasing representativeness of women is primarily due to the presence of more female external members. Women have only been appointed as external members since 2013. Since then, the proportion of women has increased, and in 2021 there were more female than male external board members.

Female board members are more common in the large farmer-owned cooperatives than in the small. A positive correlation between the size of the companies and the representation of women is identified. This is supported by large cooperatives' greater focus in annual reports and CSR strategies on women's representativeness.

Based on the cooperatives' diversity policy, their explicit and specific goals for women's representativeness on boards, interviews with board members etc. a clear awareness of the challenge of gender diversity on the boards is identified.

The article both uncovers and quantifies the problem—also in a time perspective in a case which, from a farmer-cooperative aspect, is relevant, although not fully representative in an international perspective. Such an analysis of this size has not been carried out before, and both the visibility of the problem and the mismatch between goal and result is useful for stakeholders in and around farmer-owned cooperatives.

When it comes to theoretical implications, the structural and institutional problems uncovered in the article are significant barriers to women's participation in farmer-owned cooperatives. New or alternative models and solutions must be presented and assessed. This applies, for example, to external members of the boards, who to a certain extent can solve the problems, but who also create new problems.

Specifically regarding managerial implications, challenges and unresolved issues, which necessitate managerial considerations, are identified. Explicit goals, instruments and time horizons regarding gender representation are explicitly missing in several cooperatives, which *per se* is considered to be a problem. Recognition of generally accepted goals about gender representation is an important managerial achievement. A gap between goals and results is in itself also a managerial challenge that should be solved in the short or longer term. The managerial task consists of motivating women, making board membership attractive and, not least, showing and documenting the benefits of more women on boards.

As to future research three topics deserve a deeper analysis:

First, this article is based on a case study. In order to obtain more representative conclusions, it is relevant to study other countries as well: Are the issues, goals, initiatives and results different, and what conclusions and recommendations can be drawn?

Secondly, the topic lacks more specific analyses of goals or ambitions vs. real observations for women's participation: Do farmer-owned cooperatives have goals for women's participation, are they in line with general recommendations, and how big is the gap between goal and result?

Thirdly, qualitative and quantitative studies regarding the impacts of women's participation will be both relevant and useful. It concerns both the work and management of the boards and potential impacts on the performance of the cooperative.

## Data availability statement

The raw data supporting the conclusions of this article will be made available by the authors, without undue reservation.

## Author contributions

All authors listed have made a substantial, direct, and intellectual contribution to the work and approved it for publication.

## Footnotes

^1^Personal message, September, 2022, from Christian Høegh-Andersen, Chairman of DLF amba and DLF Seeds A/S.

^2^Personal message, August 5, 2022, from Kristian Møller Sørensen, Chairman of Karup Kartoffelmelsfabrik amba and board member of KMC amba.

^3^Personal message, December 15, 2022, from Niels Dengsø Jensen, Chairman of DLG amba (Danish Farm Supply Cooperative), chairman of AP Pension, DanHatch Holding, Vilofoss etc.

## Appendix

### Names of and links to farmer-owned cooperatives in Denmark

Leverandørselskabet Danish Crown amba (CVR: 21643939).
  https://datacvr.virk.dk/enhed/virksomhed/21643939?fritekst=21643939&sideIndex=0&size=10
Danish Crown A/S (CVR: 26121264).
  https://datacvr.virk.dk/enhed/virksomhed/26121264?fritekst=26121264&sideIndex=0&size=10
Arla Foods amba (CVR: 25313763).
  https://datacvr.virk.dk/enhed/virksomhed/25313763?fritekst=25313763&sideIndex=0&size=10
DLG amba—Dansk Landbrugs Grovvareselskab (CVR: 24246930).
  https://datacvr.virk.dk/enhed/virksomhed/24246930?fritekst=24246930&sideIndex=0&size=10
Danish Agro (CVR: 59789317).
  https://datacvr.virk.dk/enhed/virksomhed/59789317?fritekst=59789317&sideIndex=0&size=10
DLF amba—Dansk Landbrugs Frøselskab (69459218).
  https://datacvr.virk.dk/enhed/virksomhed/69459218?fritekst=69459218&sideIndex=0&size=10
DLF Seeds A/S (CVR: 62556013).
  https://datacvr.virk.dk/enhed/virksomhed/62556013?fritekst=62556013&sideIndex=0&size=10
Thise Mejeri A.M.B.A. (CVR: 12425694).
  https://datacvr.virk.dk/enhed/virksomhed/12425694?fritekst=12425694&sideIndex=0&size=10
AKV Langholt amba (CVR: 34914311).
  https://datacvr.virk.dk/enhed/virksomhed/34914311?fritekst=34914311&sideIndex=0&size=10
KMC Kartoffelmelcentralen amba (CVR: 15230614).
  https://datacvr.virk.dk/enhed/virksomhed/15230614?fritekst=15230614&sideIndex=0&size=10
Kopenhagen Fur a.m.b.a. (CVR: 15275413).
  https://datacvr.virk.dk/enhed/virksomhed/15275413?fritekst=15275413&sideIndex=0&size=10
Vestjyllands Andel amba (CVR: 61729615).
  https://datacvr.virk.dk/enhed/virksomhed/61729615?fritekst=61729615&sideIndex=0&size=10
Bornholms Andelsmejeri a.m.b.a. (CVR: 30220110).
  https://datacvr.virk.dk/enhed/virksomhed/30220110?fritekst=30220110&sideIndex=0&size=10
Naturmælk amba (CVR: 17995030).
  https://datacvr.virk.dk/enhed/virksomhed/17995030?fritekst=17995030&sideIndex=0&size=10
GASA NORD GRØNT A.m.b.A (CVR: 54550715).
  https://datacvr.virk.dk/enhed/virksomhed/54550715?fritekst=54550715&sideIndex=0&size=10
Salling Grovvarer amba (CVR: 68736315).
  https://datacvr.virk.dk/enhed/virksomhed/68736315?fritekst=68736315&sideIndex=0&size=10
Vejrup Andels grovvareforening (CVR: 45231712).
  https://datacvr.virk.dk/enhed/virksomhed/45231712?fritekst=45231712&sideIndex=0&size=10
Nordsjællands Andels grovvareforening amba (CVR: 42737615).
  https://datacvr.virk.dk/enhed/virksomhed/42737615?fritekst=42737615&sideIndex=0&size=10
Gasa Odense Frugt-Grønt amba (CVR: 10639476).
  https://datacvr.virk.dk/enhed/virksomhed/10639476?fritekst=10639476&sideIndex=0&size=10
Danæg amba (CVR: 15311819).
  https://datacvr.virk.dk/enhed/virksomhed/15311819?fritekst=15311819&sideIndex=0&size=10
Andels-Kartoffelmelsfabrikken Danmark A.m.b.a. + Andels-Kartoffelmelsfabrikken Sønderjylland (AKS). (CVR: 62818328).
  https://datacvr.virk.dk/enhed/virksomhed/62818328?fritekst=62818328&sideIndex=0&size=10
Karup Kartoffelmelsfabrik A.M.B.A (CVR: 16217719).
  https://datacvr.virk.dk/enhed/virksomhed/16217719?fritekst=16217719&sideIndex=0&size=10
Andelskartoffelmelsfabrikken Midtjylland A.M.B.A. (CVR: 38569317).
  https://datacvr.virk.dk/enhed/virksomhed/38569317?fritekst=38569317&sideIndex=0&size=10
Bording Minkfodercentral A.M.B.A. (CVR: 31677513).
  https://datacvr.virk.dk/enhed/virksomhed/31677513?fritekst=31677513&sideIndex=0&size=10
Holstebro Minkfodercentral A.m.b.A. (CVR: 36582014).
  https://datacvr.virk.dk/enhed/virksomhed/36582014?fritekst=36582014&sideIndex=0&size=10
Sjællands Pelsdyrfoder (CVR: 19827186).
  https://datacvr.virk.dk/enhed/virksomhed/19827186?fritekst=19827186&sideIndex=0&size=10
Minkfodercentralen Vilsund A.M.B.A. (CVR: 55929017).
  https://datacvr.virk.dk/enhed/virksomhed/55929017?fritekst=55929017&sideIndex=0&size=10
